# Hypoxia-Induced Changes in L-Cysteine Metabolism and Antioxidative Processes in Melanoma Cells

**DOI:** 10.3390/biom13101491

**Published:** 2023-10-07

**Authors:** Leszek Rydz, Maria Wróbel, Klaudia Janik, Halina Jurkowska

**Affiliations:** Chair of Medical Biochemistry, Faculty of Medicine, Jagiellonian University Medical College, Kopernika 7 St., 31-034 Krakow, Poland; leszek.rydz@uj.edu.pl (L.R.); mtk.wrobel@uj.edu.pl (M.W.); klaudia1.janik@uj.edu.pl (K.J.)

**Keywords:** sulfane sulfur, sulfurtransferases, cystathionine-β-synthase, thioredoxin reductase-1, hypoxia, melanoma

## Abstract

This study was performed on human primary (WM115) and metastatic (WM266-4) melanoma cell lines developed from the same individual. The expression of proteins involved in L-cysteine metabolism (sulfurtransferases, and cystathionine β-synthase) and antioxidative processes (thioredoxin, thioredoxin reductase-1, glutathione peroxidase, superoxide dismutase 1) as well as the level of sufane sulfur, and cell proliferation under hypoxic conditions were investigated. Hypoxia in WM115 and WM266-4 cells was confirmed by induced expression of carbonic anhydrase IX and 6-phosphofructo-2-kinase/fructose-2,6-biphosphatase 4 by the RT-PCR and Western blot methods. It was shown that, under hypoxic conditions the inhibition of WM115 and WM266-4 melanoma cell proliferation was associated with decreased expression of thioredoxin reductase-1 and cystathionine β-synthase. These two enzymes may be important therapeutic targets in the treatment of melanoma. Interestingly, it was also found that in normoxia the expression and activity of 3-mercaptopyruvate sulfurtransferase in metastatic WM266-4 melanoma cells was significantly higher than in primary melanoma WM115 cells.

## 1. Introduction

The risk of developing melanoma is influenced by UV exposure, skin phenotype, and genetic susceptibility [1]. With a high ability and tendency to metastasize, melanoma causes the majority of skin cancer-related deaths [2]. Melanocyte cells are in constant exchange with surroundings (mainly keratinocytes and fibroblasts) creating a specific microenvironment [3]. Melanocytes reside in the dermal–epidermal junction in human skin in a hypoxic environment [4]. Hypoxic conditions affect cancer-associated fibroblast behaviour, extracellular matrix rearrangement, blood vessel formation, and immune cell activity [5,6,7,8]. Cellular adaptation to a hypoxic environment is enhanced by the expression of hypoxia-inducible factor-1 and -2 (HIF-1, HIF-2) [4,9]. HIF-1 is a heterodimer that consists of HIF-1α and HIF-1β subunits [8]. Under normoxic conditions, the HIF-1α subunit undergoes hydroxylation leading to subunit degradation and a loss of activity [7]. On the other hand, oxygen absence has a rapid impact on increasing HIF-1α subunit mRNA expression and protein levels [10]. Hydroxylation and final degradation are inhibited under low oxygen conditions [7]. Under hypoxia, HIF dimer is accumulated, then it is translocated to the nucleus [4,11,12]. HIF-dependent activity modulates key gene expression responsible for metabolism, pH homeostasis, autophagy, angiogenesis, and metastasis [4,13]. The transcription factor HIF-1 is a key regulator of carbonic anhydrase IX (CAIX) and phosphofructo-2-kinase/fructose-2,6-biphosphatase 4 (PFKFB4) expression [14,15]. PFKFB4 and CAIX expression is upregulated during hypoxia in melanoma-derived cancer cells [16,17,18].

Reactive oxygen species (ROS) accumulation leads directly to oxidative damage and their accumulation is associated with obesity, diabetes, cancers, aging, and other age-related diseases [19]. To nullify ROS’s negative impact on mitochondrial and cellular components, an effective defense mechanism exists. It includes mitochondrial or cytosolic superoxide dismutase (SOD) disproportionate O^2−^ to H_2_O_2_. Hydrogen peroxide is efficiently removed by catalase and peroxiredoxin/glutathione peroxidase (GPx) [20]. Another essential and ubiquitous component of antioxidant defense is the thioredoxin/thioredoxin reductase system [21]. It consists of thioredoxin (TRX), thioredoxin reductase (TXNRD1), and NADPH, which is another antioxidative defense line. TXNRD1 is found in mitochondria whereas TXNRD2 is located in cytoplasm [22]. TRX usually interacts with a broad spectrum of target proteins utilizing thiol–disulfide exchange [21]. TXNRD containing cysteine and selenocysteine in the active site provides electrons directly to TRX using electrons from NADPH [22].

A hypoxic environment may stimulate ROS production by impairing the mitochondrial respiratory chain functioning [19]. However, there could be more sources of ROS such as NADPH oxidase or xanthine oxidase [23]. Hernansanz-Agustin et al. [24] showed that acute mild hypoxia results in a rapid burst of ROS when it occurs, but it diminishes with time. A prolongated state of hypoxia results in the accumulation of reduced nicotinamide adenine dinucleotide phosphate (NADH) inside the mitochondria which can be oxidized by Complex I to propel the mitochondrial transport chain, additionally generating ROS [25]. Temporary hypoxia is dangerous because of ROS, the generation of which is increased especially during the cyclic hypoxia/re-oxygenation process. Accumulation of ROS could lead to necrosis [19].

Primary (WM115) and metastatic (WM266-4) cutaneous melanoma cells were developed in the same individual [26]. Cancer progression may be correlated with an increase in the expression of certain proteins [27]. A comparison of their proteome showed numerically more proteins in the WM266-4 cell line, indicating increased demands connected with the process of metastasis [28]. Researchers provided detailed information about the proteome of these cancer cell lines [28,29].

Sulfane sulfur which can directly eliminate ROS plays an essential role in the antioxidative system in cells [30,31]. It exists in different forms in cells (persulfides–RSSH; polysulfides–R-S_x_-R, n > 3) or within a biological continuum with hydrogen sulfide (H_2_S) [32,33,34]. Sulfane sulfur-containing compounds can be produced endogenously by several enzymes including 3-mercaptopyruvate sulfurtransferase (MPST), cystathionine γ-lyase (CTH), and thiosulfate sulfurtransferase (TST) as well as cystathionine β-synthase (CBS) [35,36].

This study aimed to determine the effect of a hypoxic environment on expression/activity enzymes involved in L-cysteine metabolism and sulfane sulfur-containing compound formation, as well as on the antioxidant defense system of two related melanoma cell lines (WM115 and WM266-4).

## 2. Materials and Methods

### 2.1. Cell Culture

The human melanoma cell lines WM115 and WM 266-4 were a gift from the Department of Glycoconjugate Biochemistry (Jagiellonian University, Institute of Zoology and Biomedical Research, Krakow, Poland) and the cell lines were obtained from the ESTDAB Melanoma Cell Bank (Tübingen, Germany). Cells were cultured using RPMI Medium 1640 with L-Glutamine (ThermoFisher Scientific, Waltham, MA, USA), and supplemented with 10% of fetal bovine serum (EURx, Gdańsk, Poland) and antibiotic mix (100 U/mL of penicillin and 100 µg/mL of streptomycin) (ThermoFisher Scientific, Waltham, MA, USA). Cells were cultured in standard conditions (21% oxygen, hereafter referred to as normoxia) or in the Modular Incubator Chamber (Billups-Rothenberg, Inc., San Diego, CA, USA; 1% O_2_, 5% CO_2_, 94% N_2_, hereafter referred to as hypoxia). To obtain hypoxic conditions, cells were maintained as described by Kocemba-Pilarczyk et al. [37]. Cells were maintained at 37 °C in 5% CO_2_ and 95% humidity.

### 2.2. Cell Proliferation Measurement

WM115 or WM266-4 cells were suspended in a freshly prepared medium supplemented as mentioned above (Section 2.1). Cells were seeded on a 6-well plate (NEST Biotechnology, Jiangsu, China) at a density of 2.5 × 10^5^/well. After 24 h the medium was changed and the cells were further cultured using an environment with different oxygen presence as mentioned above in Section 2.1. Cell proliferation was examined using the modified crystal violet method by Gillies et al. [38]. The absorbance was measured using an Epoch Microplate Spectrophotometer (BioTek Instruments, Inc., Winooski, VT, USA).

### 2.3. Expression of Hypoxia Markers (CAIX, PFKBF4), and Proteins Involved in L-cysteine Metabolism (CBS, MPST, CTH, TST) and Thioredoxin/Glutathione Systems (TXNRD1, TRX, GPx)

#### 2.3.1. Total RNA Isolation

Total cellular RNA was extracted from cells using TRIzol reagent according to the manufacturer’s instructions (Invitrogen, Carlsbad, CA, USA). Quantification and examination of the quality of the RNA were performed using a NanoDrop ND-1000 Spectrophotometer (NanoDrop Technologies, Wilmington, DE, USA). The quality of the RNA samples was determined by spectrophotometric analysis of the ratio: 260 nm/280 nm absorbance. Obtained RNA solutions were stored at −80 °C until further procedures.

#### 2.3.2. Reverse Transcription (RT)

A total of 2 µg of isolated RNA was used to obtain cDNA by using the NG-dART RT kit (#E0801-01, EURx, Gdańsk, Poland) following the manufacturer’s protocol. A working solution composed of dART Reverse Transcriptase (NG dART RT Mix, EURx, Gdańsk, Poland), with MgCl_2_, RNAse inhibitor, and dNTP mix was prepared. The working solution (6 µL) was mixed with a total of 14 µL of properly diluted RNA (20 µL of the final volume of the reaction mixture). Then, samples were incubated according to the manufacturer’s protocol for 10 min at 25 °C, 50 min at 65 °C, and 5 min at 85 °C. The obtained cDNA was stored at −20 °C until further procedures.

#### 2.3.3. Polymerase Chain Reaction (PCR)

cDNA level was measured for the genes: *CAIX*, *PFKFB4*, *CTH*, *CBS*, *TST*, *MPST*, *TXNRD1*, *TRX*, *GPx*, and *β-actin*. The PCR reaction was performed using a Color OptiTaq PCR Master Mix (2x) kit (#E2915-01 EURx, Gdańsk, Poland) following the manufacturer’s protocol. The working solution was prepared up to a total of 25 µL containing: 1.25 U OptiTaq DNA Polymerase, 1X reaction buffer (1.5 mM MgCl_2_), 0.25 mM of each dNTP, 0.2 µM of each gene-specific primer pair, and nuclease-free water. The specific primers (Sigma-Aldrich, Saint Louis, MO, USA) of the tested genes are listed in Table 1. The working buffer was mixed with 2 µL of synthesized cDNA. Cycling conditions were strictly specific for different genes and are listed below (Table 2).

#### 2.3.4. Densitometric Evaluation of PCR Products

The PCR-amplified products were separated by gel electrophoresis on 2.0% agarose gel containing ethidium bromide staining. The bands were visualized under UV light and photographed using the ChemiDocTM MP Imaging System (Bio-Rad, Hercules, CA, USA). The intensity of the band was measured by densitometric analysis using the dedicated computer system UVI-KS 4000i/ImagePC (Syngen Biotech, Wrocław, Poland). β-actin was used as a housekeeping gene to normalize all samples.

### 2.4. Western Blot Analysis

The cells were lysed in a buffer (50 mM Tris–HCl, pH 7.5, 150 mM NaCl, 1 mM EDTA, 0.5% NP-40) and supplemented with 1X Complete Protease Inhibitor Cocktail (Sigma-Aldrich Corp., St. Louis, MO, USA). Cell lysates were centrifuged at 20,000× *g* for 15 min at 4 °C. The concentration of proteins was measured using the BCA protein assay (Thermo Scientific/Pierce Biotechnology, Rockford, IL, USA). A total of 25 µg of each lysate were separated on a 12% SDS-polyacrylamide gel and then transferred to 0.22 µm PVDF membranes (Bio-Rad, Hercules, CA, USA). Subsequently, the membranes were blocked in 5% non-fat milk for 1 h, and incubated with specific primary antibodies overnight at 4 °C: anti-CBS (1:800; mouse monoclonal, #H00000875-MO1, Abnova, Taiwan), anti-TST (1:800; mouse monoclonal, #66018-1-Ig, Proteintech Group, Rosemont, IL, USA), anti-MPST (1:800; rabbit polyclonal, #GTX108274, GeneTex, Inc., Irvine, CA, USA), anti-CTH (1:1000; mouse monoclonal, #60234-1-Ig, Proteintech Group, Rosemont, IL, USA), anti-TRX (1:1000; rabbit polyclonal, #14999-1-AP, Proteintech Group, Rosemont, IL, USA), anti-TXNRD1 (1:500 rabbit polyclonal, #11117-1-AP, Proteintech Group, Rosemont, IL, USA), anti-CAIX (1:1000; rabbit polyclonal, #A13682, ABclonal, Woburn, MA, USA), anti-SOD1 (1:1000; rabbit polyclonal, #10269-1-AP, Proteintech Group, Rosemont, IL, USA), and anti-β-actin (1:1000; mouse monoclonal, #60008-1-Ig, Proteintech Group, Rosemont, IL, USA). Then, the membranes were washed three times in T-TBS 1X, incubated with a goat anti-rabbit alkaline phosphatase-conjugated secondary antibodies (1:2000, Proteintech Group, Rosemont, IL, USA), and reacted with nitro blue tetrazolium and 5-bromo-4-chloro-3-indolyl phosphate (NBT-BCIP) stock solution (Roche Applied Science, Penzberg, Germany). β-actin was used as the internal control. Densitometric analysis of proteins was performed using the ChemiDocTMMP Imaging System (Bio-Rad, Hercules, CA, USA).

### 2.5. Homogenate Preparation for Biochemical Measurements

Cells were washed and suspended in 0.1 M phosphate buffer pH 7.5, in the ratio of 1 × 10^6^ cells/0.04 mL of the buffer and sonicated 3 × 5 s at 4 °C (Bandelin Sonoplus GM 70, Hannover, Germany). After centrifugation at 4500× *g* at 4 °C for 10 min., the supernatant was used for the determination of various biochemical measurements: the activity of MPST, and the levels of sulfane sulfur and protein.

### 2.6. MPST Activity Measurement

MPST activity was determined by the method of Valentine and Frankelfeld [46], according to the procedure described by Wróbel et al. [47]. Firstly, the incubation mixture was composed of 250 µL 0.12 M sodium phosphate buffer (pH 8.0), 50 µL 0.5 M sodium sulfite (Sigma-Aldrich), 50 µL 0.15 M DL-Dithiothreitol (DTT, Sigma-Aldrich), 50 µL distilled water, 50 µL 0.1 M sodium salt of 3-mercaptopyruvate acid (Santa Cruz Biotechnology, Dallas, TX, USA), and 50 µL homogenate. The total volume of 500 µL was incubated for 15 min. Then, 250 µL of 1.2 M perchloric acid (PCA, POCh S.A., Gliwice, Poland) was used to stop the reaction. The samples were centrifuged at 1600× *g* for 5 min. A total of 100 µL of the supernatant was transferred to a mixture containing 1200 µL 0.12 M sodium phosphate buffer (pH 8.0), 100 µL 0.1 M N-ethylmaleimide (Sigma-Aldrich), and 50 µL 5 mg/mL β-Nicotinamide adenine dinucleotide reduced disodium salt (NADH, Sigma-Aldrich). After equilibration at 37 °C, 2.5 µL (7 IU) L-lactic dehydrogenase (Sigma-Aldrich) was added and the absorbance was measured at 340 nm. The enzymatic activity was calculated as µmoles of pyruvate produced during the 1 min incubation at 37 °C per 1 mg of protein.

### 2.7. Sulfane Sulfur Level

Sulfane sulfur level was determined by the method of Wood et al. [48]. The method is based on a cold cyanolysis reaction which allows colorimetric detection of ferric thiocyanate complex. The sulfane sulfur level was calculated as µmoles of SCN produced per 1 mg of protein.

### 2.8. Protein Level

The total protein content was determined by the method of Lowry et al. [49]. The method is based on the two-step reaction of peptide bonds and aromatic amino acids contained in proteins with the Folin–Ciocalteu reagent (Sigma-Aldrich) in an alkaline environment in the presence of copper ions.

### 2.9. Statistical Analysis

Statistical analyses were performed using GraphPad Prism 9.0 (GraphPad Software Inc., La Jolla, CA, USA). The results were presented as the means ± standard deviations (SD). Each data set was analyzed by the Mann–Whitney test or Student’s *t*-test, with values of *p* < 0.05 as statistically significant. The results show the outcome of at least three independent experiments.

## 3. Results

### 3.1. Hypoxia-Related Genes Expression in Two Melanoma Cell Lines (WM115, WM266-4) under Different Oxygen Concentrations

**Figure 1:**
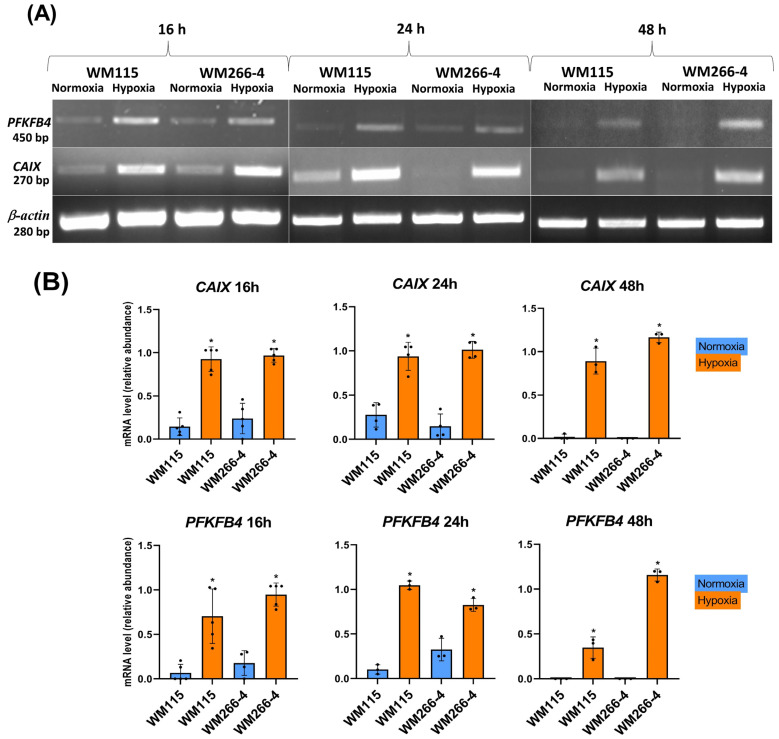
The expression of PFKFB4, CAIX, and β-actin on the mRNA level in human melanoma WM115 and WM266-4 cell lines under normoxia and hypoxia (RT-PCR). Experiments were performed at least three times with similar results. Representative results of mRNA expression (**A**); and densitometry analysis of obtained gels (**B**). Original images of RT-PCR and Western blot results are shown in Supplementary Materials. Quantification of gene expressions (mRNA level) was conducted via the analysis of gels normalized by using β-actin as the internal control (**B**). The results are expressed as mean ± SD. Stars indicate significant differences between normoxia and hypoxia (* *p* ≤ 0.05). Experiments were performed at least three times. Each dot (●) represents an individual measurement.

**Figure 2:**
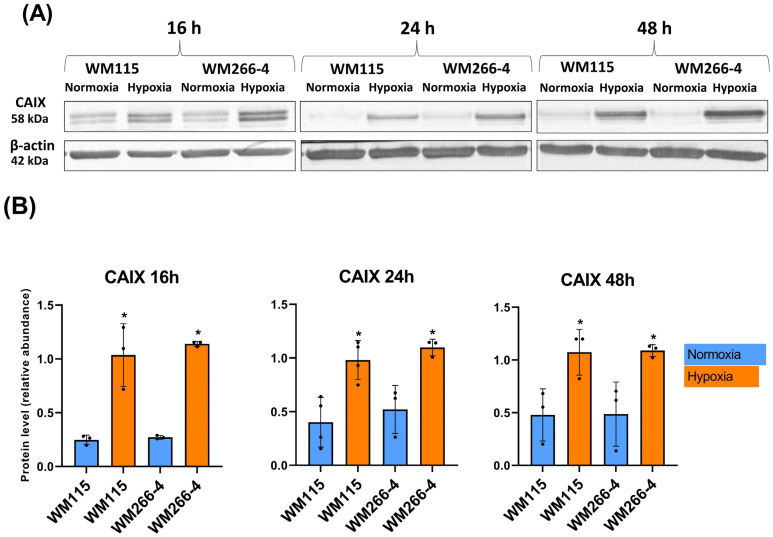
The expression of CAIX, and β-actin on the protein level in human melanoma WM115 and WM266-4 cell lines under normoxia and hypoxia (Western blot). Experiments were performed at least three times with similar results. Representative results of protein level (**A**); and densitometry analysis of obtained gels (**B**). Original images of RT-PCR and Western blot results are shown in Supplementary Materials. Quantification of gene expressions (protein level) was conducted via the densitometry analysis of gels (**B**). The results are expressed as mean ± SD. Stars indicate significant differences between normoxia and hypoxia (* *p* ≤ 0.05). Experiments were performed at least three times. Each dot (●) represents an individual measurement.

We initially analyzed the response of WM115 and WM266-4 cell lines to low oxygen concentration levels. To confirm the hypoxic condition we have evaluated the expression of carbonic anhydrase IX (CAIX) and phosphofructo-2-kinase/fructose-2,6-biphosphatase 4 (PFKFB4) using RT-PCR (Figure 1A,B). To further confirm our findings we have analyzed CAIX protein levels using Western blot (Figure 2A,B). We have conducted our experiments in consecutive time points (16, 24, 48 h). A hypoxic environment was established at each mentioned time point. The representative results are shown in Figure 1A (RT-PCR) with the following densitometry analysis of obtained bands (Figure 1B), Western blot analysis (Figure 2A), and with the following densitometry analysis of obtained bands (Figure 2B).

### 3.2. The Effect of a Hypoxic Environment on Human Melanoma WM115 and WM266-4 Cells Proliferation

**Figure 3:**
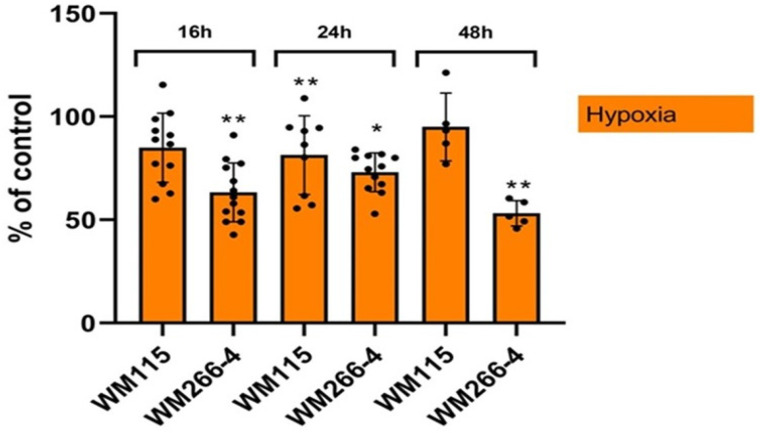
The influence of reduced oxygen presence on human melanoma cell line (WM115 and WM266-4) proliferation. Cells were incubated under normoxia and hypoxia for 16, 24, and 48 h. Cell proliferation was analyzed using crystal violet staining. The results are expressed as mean ± SD. Stars indicate significant differences between normoxia and hypoxia (results are expressed as % of control); * *p* ≤ 0.05; ** *p* ≤ 0.01. Experiments were performed at least three times. Each dot (●) represents an individual measurement.

The human melanoma WM115 and WM266-4 cell lines were cultured under normoxic and hypoxic conditions for 16, 24, and 48 h. As shown in Figure 3, the proliferation of both WM115 and WM266-4 cells was statistically lower after 24 h under hypoxic conditions. Moreover, WM266-4 cells proliferation was lower under hypoxia compared to the normoxic environment at all time points. WM115 cells proliferation decreased significantly only after 24 h of incubation with low oxygen concentration.

### 3.3. The Effect of Hypoxia Environment on Sulfane Sulfur Level in Human Melanoma WM115 and WM266-4 Cells

**Figure 4:**
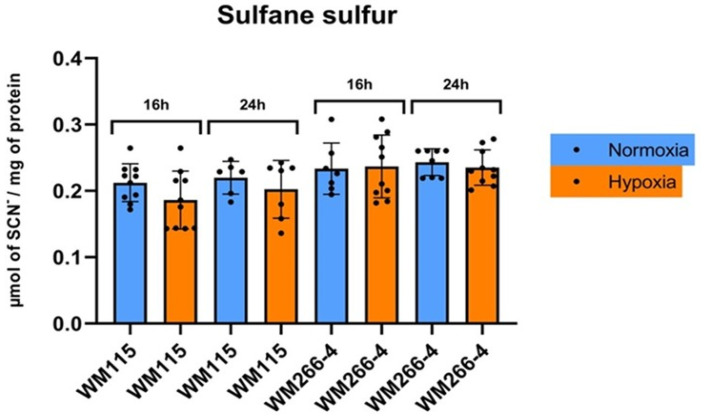
The influence of reduced oxygen presence on human melanoma cell lines’ (WM115 and WM266-4) sulfane sulfur level. Cells were incubated under normoxia and hypoxia for 16, 24, and 48 h. The results are expressed as mean ± SD. Experiments were performed at least three times. Each dot (●) represents an individual measurement.

The results presented in Figure 4 showed no changes in sulfane sulfur level at each time point between normoxia and hypoxia in melanoma both WM115 and WM266-4 cells.

### 3.4. The Expression and Activity of 3-Mercapropyruvate Sulfurtransferase in Human Melanoma WM115 and WM266-4 Cells in a Hypoxia Environment

**Figure 5:**
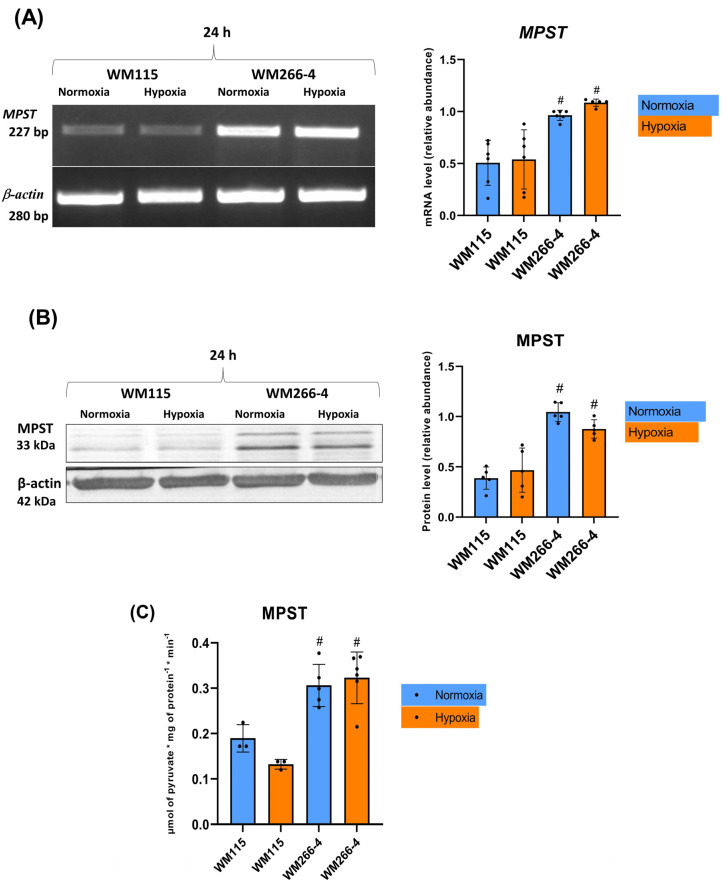
The effect of hypoxia on MPST expression and activity: (**A**) mRNA level, (**B**) protein level, and (**C**) enzymatic activity in human melanoma cell lines (WM115 and WM266-4). Cells were incubated under normoxia and hypoxia for 24 h. Representative results of MPST mRNA (**A**) and protein (**B**) gel/blot are shown in Figure 5. Original images of RT-PCR and Western blot results are shown in Supplementary Materials. Quantification of MPST expressions (mRNA level and protein level) was performed via the densitometry analysis of gels (**A**,**B**). The results are expressed as mean ± SD. # indicates significant differences between parallel conditions (e.g., WM115 normoxia vs. WM266-4 normoxia; # *p* ≤ 0.05). Experiments were performed at least three times. Each dot (●) represents an individual measurement.

There were no differences in melanoma cells (WM115 and WM266-4) regarding mRNA expression, protein level, and enzyme activity of MPST under hypoxic conditions compared to normoxia (Figure 5A–C). WM266-4 cells showed significantly greater levels of mRNA and protein, as well as activity of MPST, compared to WM115 cells under both normoxic and hypoxic conditions (Figure 5A–C).

### 3.5. The Expression of Other Enzymes Involved in L-cysteine Metabolism in Human Melanoma WM115 and WM266-4 Cells in a Hypoxia Environment

**Figure 6:**
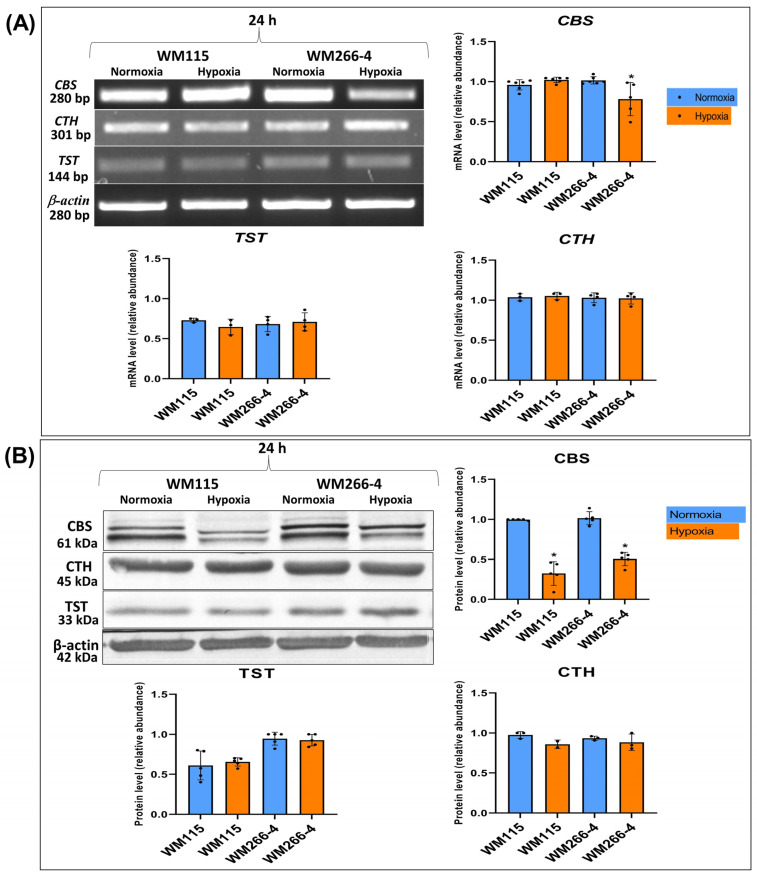
The effect of hypoxia on CBS, CTH, and TST expression: (**A**) mRNA level, and (**B**) protein level in human melanoma cell lines (WM115 and WM266-4). Cells were cultured under normoxia and hypoxia for 24 h. Representative results of particular gene mRNA (**A**) and protein (**B**) gels/blots are shown in Figure 6. Original images of RT-PCR and Western blot results are shown in Supplementary Materials. Quantification of gene expressions (mRNA level and protein level) was performed via the densitometry analysis of gels (**A**,**B**). The results are expressed as mean ± SD. Stars indicate significant differences between normoxia and hypoxia (* *p* ≤ 0.05). Experiments were performed at least three times. Each dot (●) represents an individual measurement.

Figure 6A,B show the expression of CBS, CTH, and TST on mRNA and protein levels. Under hypoxic conditions, expression of CBS on protein level was significantly reduced in both melanoma WM115 and WM266-4 cells after 24 h of culture in hypoxia (Figure 6B). No changes were indicated in CTH and TST expression in the same culture conditions.

### 3.6. The Expression of Enzymes Involved in Antioxidative Cellular Defense in Human Melanoma WM115 and WM266-4 Cells in a Hypoxia Environment

**Figure 7:**
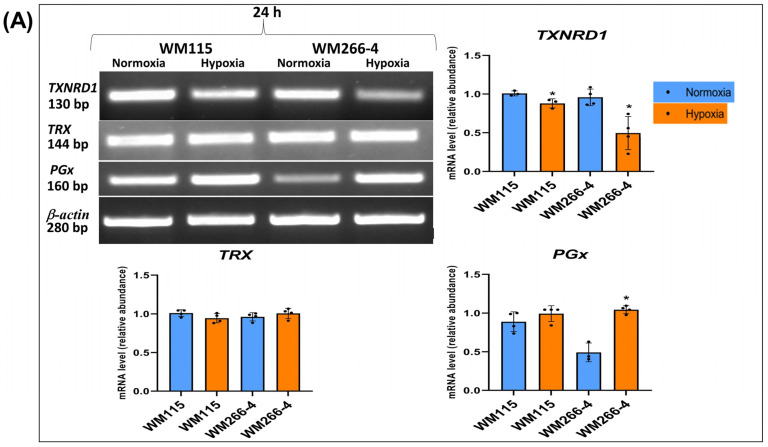

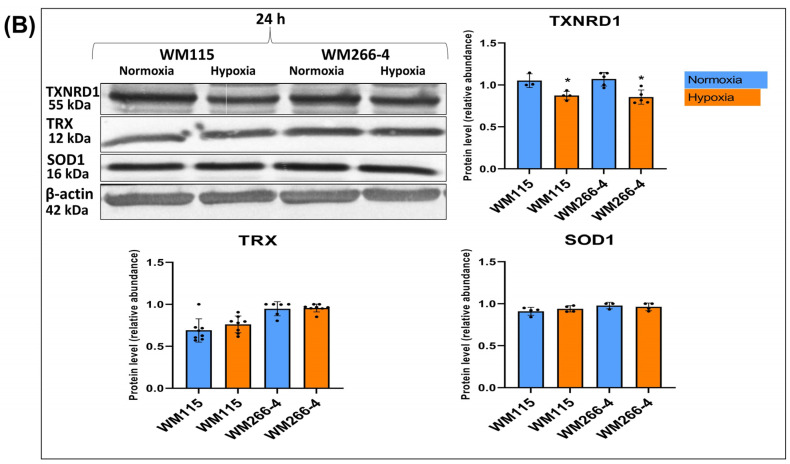
The effect of hypoxia on TXNRD1, TRX, PGx, and SOD1 expression: (**A**) mRNA level and (**B**) protein level in human melanoma cell lines (WM115 and WM266-4). Cells were cultured under normoxia and hypoxia for 24 h. Representative results of particular gene mRNA (**A**) and protein (**B**) gels/blots are shown in Figure 7. Original images of RT-PCR and Western blot results are shown in Supplementary Materials. Quantification of gene expressions (mRNA level and protein level) was performed via the densitometry analysis of gels (**A**,**B**). The results are expressed as mean ± SD. Stars indicate significant differences between normoxia and hypoxia (* *p* ≤ 0.05). Experiments were performed at least three times. Each dot (●) represents an individual measurement.

Figure 7A,B show the expression of TXNRD1, TRX, PGx, and SOD1 on mRNA/protein levels. Under the hypoxic condition, the expression of mRNA and the protein level of thioredoxin reductase-1 were significantly reduced in both melanoma WM115 as well as in WM266-4 cells after 24 h of culture in a low oxygen environment (Figure 7A,B). Results also showed a significant enhancement of glutathione peroxidase expression in WM266-4 cells (Figure 7A). No changes were found in the expression of thioredoxin (Figure 7A,B) and superoxide dismutase 1 (Figure 7B) under hypoxia.

## 4. Discussion

In this research paper, we examined the expression levels of sulfurtransferases (MPST, TST, CTH) and cystathionine beta-synthase in human primary (WM115) and metastatic (WM266-4) melanoma cell lines both in normoxic and hypoxic conditions. Interestingly, we have found that in WM266-4 cells cultured in normoxic conditions, the expression of MPST, (Figure 5A,B) as well as the activity of MPST (Figure 5C), were significantly higher when compared to WM115 cells. Thus, it seems that MPST can play an important role in the progression of human melanoma cells (WM115 and WM266-4) compared to other sulfurtransferases such as TST and CTH, of which, expression in both cell lines was not changed. Under the same normoxic culture conditions, the level of sulfane sulfur in these cells was comparable (Figure 4).

In our studies, the induction of hypoxia in WM115 and WM266-4 melanoma cells during culture was confirmed by increasing the mRNA as well as protein levels of carbonic anhydrase IX, and additionally by increasing the mRNA level of 6-phosphofructo-2-kinase/fructose-2,6-biphosphatase 4 (Figure 1A,B and Figure 2A,B), which was also reported previously by Trojan et al. [44].

The results of the present study demonstrated that under hypoxic conditions the inhibition of WM115 and WM266-4 melanoma cell proliferation (Figure 3) is associated with decreased thioredoxin reductase 1 on both mRNA and protein levels (Figure 7A,B). Hypoxic stress can cause the overproduction of reactive oxygen species, which can inhibit the activity of antioxidant enzymes such as thioredoxin reductase 1 [50]. Suvei et al. [51] reported that CoCl_2_-induced hypoxia decreased the viability of human melanoma cells (A2058 and G361), increased ROS production, and induced cell apoptosis. Reduction in thioredoxin reductase 1 of both mRNA and protein levels was observed in EM56 and DT cells [50]. Naranjo-Suare et al. [50] found that under hypoxic conditions, thioredoxin reductase 1 deficient cells showed a larger accumulation of ROS compared to control cells, whereas thioredoxin reductase 1 overexpression was able to block the hypoxic generation of ROS.

Interestingly, we have also observed that the cystathionine beta-synthase protein level in hypoxia was decreased in both WM115 and WM266-4 cells (Figure 6A,B). At the same time, the mRNA and protein levels of MPST, CTH, and TST remained unchanged. Utilizing hypoxic conditions, the study performed on two different human ovarian cancer cell lines (ES and OVCAR3) derived from two different histological types, did not observe differences in MPST protein levels, although the content of mitochondrial MPST was higher compared to the cytosolic fraction [52]. A decrease in the level of CBS with no changes in the level of MPST and CTH under hypoxic conditions was found in human umbilical vein endothelial cells (HUVECs) and human aortic endothelial cells (HAOEC) [53]. The authors showed that with a reduced level of CBS under hypoxic conditions, the level of H_2_S also decreased and the proliferation of endothelial cells was inhibited [53]. It was found that the endogenous level of H_2_S was markedly reduced upon hypoxic stress generated by Na_2_S_2_O_4_ in human neuroblastoma cells (SH-SY5Y), and CBS over-expression attenuated hypoxia-induced cell apoptosis [54].

The role of cystathionine beta-synthase in many tissues during hypoxia is unclear— CBS could play protective and detrimental effects. Changes in the expression of CBS in hypoxia conditions depend on the organism, tissue, or subcellular localization [55]. Based on our results, we can say that in melanoma cell lines such as WM115 and WM266-4 cells, the downregulation of CBS, as well as thioredoxin reductase 1 expression, may play a significant role in inhibiting cell proliferation. Reduction of CBS expression will result in decreasing formation of L-cysteine from methionine, leading to lower glutathione level and cellular proliferation.

We observed that the level of sulfane sulfur determined in melanoma WM115 and WM266-4 cells after 16 and 24 h of culture under hypoxic conditions was unchanged compared to normoxia (Figure 4). Gao et al. [56] reported that there is a close relationship between sulfane sulfur and hypoxia in living cells and in vivo. However, the overall level of sulfane sulfur is affected by the degree and length of hypoxic stress.

Our research showed an increase in the expression of glutathione peroxidase in metastatic WM266-4 cells in hypoxia (Figure 7A) while the expression of superoxide dismutase 1 was not changed (Figure 7B). Cancer cells have increased ROS levels and thus upregulate the antioxidant response, including glutathione peroxidases [57]. Peng et al. [58] showed that glutathione peroxidase was upregulated in human pancreatic (Panc-1) and Panc-1 cancer stem-like cells (Panc-1 CSCs) after exposure to hypoxia. Hypoxia reduces the concentration of glutathione in the hepatic Hep3B and HEK293 kidney cell lines by the reduction in cystine uptake [59]. Ros-BulloÂn et al. reported [60] that whole blood glutathione peroxidase activity in melanoma patients was significantly decreased when compared with that in healthy people. In melanoma cell lines (A375, SK-MEL-1, SK-MEL-2, and SK-MEL-24), it was found [61] that the mRNA and protein expressions of glutathione peroxidase 3 were significantly lower than that in normal human skin melanocyte PIG1 cells. Overexpression of glutathione peroxidase 3 inhibited the viability of human melanoma A375 cells and tumor growth [61].

## 5. Conclusions

Interestingly, it was found that in human metastatic WM266-4 melanoma cells the expression of MPST (on the mRNA and protein level) as well as the activity of MPST were significantly higher than in primary melanoma WM115 (both cell lines were developed from the same individual). Therefore, it seems that modulating the expression/activity of MPST in metastatic melanoma cells by administering compounds that inhibit its activity may give promising results in further studies. Hypoxia reduces the expression (mRNA/protein level) of cystathionine beta-synthase as well as thioredoxin reductase 1 and inhibits the proliferation of WM115 and WM266-4 melanoma cells. Thus, both CBS and TXNRD1 may also be important therapeutic targets in the treatment of melanoma.

## Figures and Tables

**Table 1:** The primer sequence used for RT-PCR.

Gene	Forward	Reverse	RT-PCR Product Size (bp)	Reference
*TRX*	5′-GGT GAA GCA GAT CGA GAG CA-3′	5′-TCA TTT TGC AAG GCC CAC AC-3′	144 bp	[NCBI database]
*TXNRD1*	5′-ACG TTA CTT GGG CAT CCC TG-3′	5′-AGA AAT CCA GCG CAC TCC AA-3′	130 bp	[NCBI database]
*GPx*	5′-ACA CCC AGA TGA ACG AGC TG-3′	5′-AGC ATG AAG TTG GGC TCG AA-3′	160 bp	[NCBI database]
*MPST*	5′-CCA GGT ACC GTG AAC ATC CC-3′	5′-TGT ACC ACT CCA CCC AGG A-3′	227 bp	[39]
*CBS*	5′-CGC TGC GTG GTC ATT CTG CC-3′	5′-TCC CAG GAT TAC CCC CGC CT-3′	280 bp	[40]
*CTH*	5′-GCA AGT GGC ATC TGA ATT TG-3′	5′-CCC ATT ACA ACA TCA CTG TGG-3′	301 bp	[41]
*TST*	5′-CCA GCT GGT GGA TTC AAG GT-3′	5′-CCC TTC TCG AAG CCA TCC TC-3′	144 bp	[42]
*β-actin*	5′-CTG TCT GGC GGC ACC ACC AT-3′	5′-GCA ACT AAG TCA TAG TCC GC-3′	254 bp	[43]
*CAIX*	5′-TAC AGC TGA ACT TCC GAG CG-3′	5′-CTA GGC TCC AGT CTC GGC TA-3′	270 bp	[44]
*PFKFB4*	5′-GGG ATG GCG TCC CCA CGG G-3′	5′-CGC TCT CCG TTC TCG GGT G-3′	450 bp	[44]

**Table 2:** The conditions of PCR for specific gene fragment amplification.

Gene	Initiation	Denaturation	Amplification	Elongation	Termination	Reference
*TRX*	5 min at 94 °C	30 s at 94 °C	30 s at 56 °C	1 min at 72 °C for 27 cycles	8 min at 72 °C	first time published
*TXNRD1*	5 min at 94 °C	30 s at 94 °C	30 s at 56 °C	1 min at 72 °C for 28 cycles	8 min at 72 °C	first time published
*GPx*	5 min at 94 °C	30 s at 94 °C	30 s at 56 °C	1 min at 72 °C for 29 cycles	8 min at 72 °C	first time published
*MPST*	5 min at 94 °C	30 s at 94 °C	30 s at 56 °C	2 min at 72 °C for 28 cycles	8 min at 72 °C	[39]
*CBS*	5 min at 94 °C	30 s at 94 °C	30 s at 60 °C	2 min at 72 °C for 38 cycles	8 min at 72 °C	[40]
*CTH*	5 min at 94 °C	30 s at 94 °C	60 s at 51 °C	8 min at 72 °C for 28 cycles	10 min at 72 °C	[45]
*TST*	5 min at 94 °C	30 s at 94 °C	30 s at 65.2 °C	1 min at 72 °C for 28 cycles	8 min at 72 °C	[42]
*β-actin*	5 min at 94 °C	30 s at 94 °C	30 s at 54 °C	2 min at 72 °C for 28 cycles	8 min at 72 °C	[45]
*CAIX*	5 min at 95 °C	30 s at 95 °C	30 s at 58 °C	30 s at 72 °C for 27 cycles	10 min at 72 °C	[44]
*PFKFB4*	5 min at 95 °C	30 s at 95 °C	30 s at 58 °C	30 s at 72 °C for 27 cycles	10 min at 72 °C	[44]

## Data Availability

The data presented in this study are available on request from the corresponding author.

## Supplementary Materials

The following supporting information can be downloaded at: https://www.mdpi.com/2218-273X/13/10/1491/s1. File S1: Original images of RT-PCR and Western blot analysis (expression on mRNA and protein levels).
