# Supplementary material for: Hypoxia-Induced Changes in L-Cysteine Metabolism and Antioxidative Processes in Melanoma Cells
Source: Biomolecules. 2023 Oct 7;13(10):1491. doi: 10.3390/biom13101491 (PMC10604596; doi:10.3390/biom13101491)
Supplement: Supplementary file 1 [file biomolecules-13-01491-s001.zip › biomolecules-2616374-supplementary.pdf]

Supplementary Materials - Original images of RT-PCR and Western blot analysis (expression on mRNA and proteins levels).

RT-PCR

1 2 3 4 5

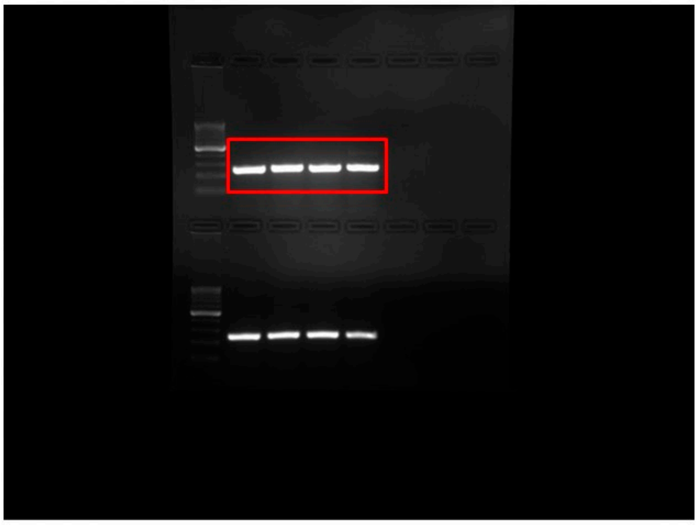

β-actin

1. Molecular weight DNA ladder
2. WM115, Normoxia, 16h
3. WM115, Hypoxia, 16h
4. WM266-4, Normoxia, 16h
5. WM266-4, Hypoxia, 16h

RT-PCR

1 2 3 4 5

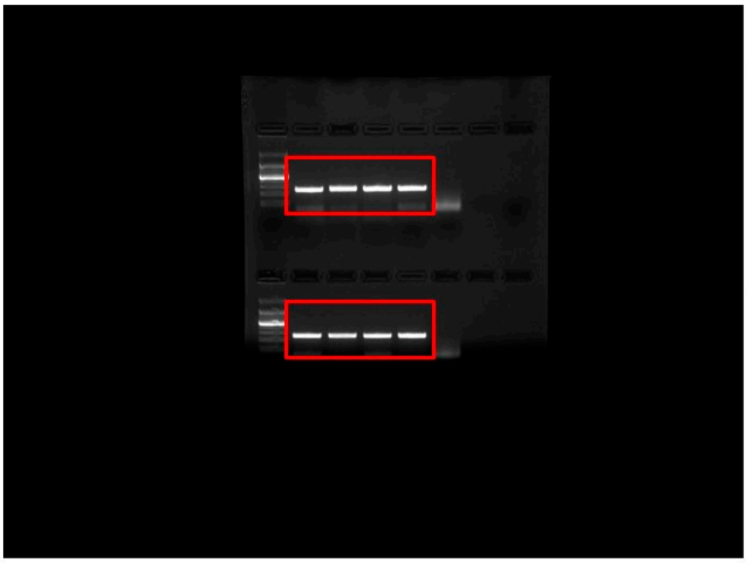

β-actin

<u>Upper gel</u>
1. Molecular weight DNA ladder
2. WM115, Normoxia, 24h
3. WM115, Hypoxia, 24h
4. WM266-4, Normoxia, 24h
5. WM266-4, Hypoxia, 24h

<u>Lower gel</u>
1. Molecular weight DNA ladder
2. WM115, Normoxia, 48h
3. WM115, Hypoxia, 48h
4. WM266-4, Normoxia, 48h
5. WM266-4, Hypoxia, 48h

RT-PCR

Carbonic anhydrase IX

1 2 3 4 5

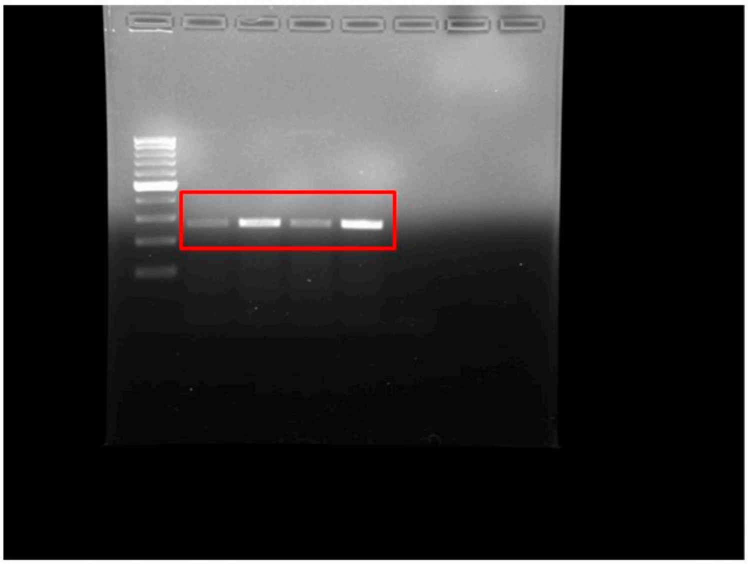

CAIX

1. Molecular weight DNA ladder
2. WM115, Normoxia, 16h
3. WM115, Hypoxia, 16h
4. WM266-4, Normoxia, 16h
5. WM266-4, Hypoxia, 16h

RT-PCR

Carbonic anhydrase IX

1 2 3 4 5

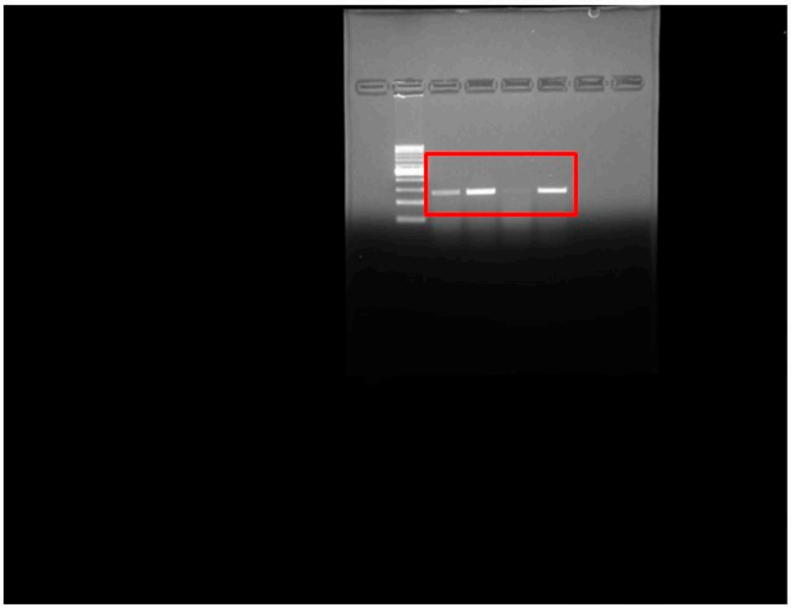

CAIX

1. Molecular weight DNA ladder
2. WM115, Normoxia, 24h
3. WM115, Hypoxia, 24h
4. WM266-4, Normoxia, 24h
5. WM266-4, Hypoxia, 24h

RT-PCR

Carbonic anhydrase IX

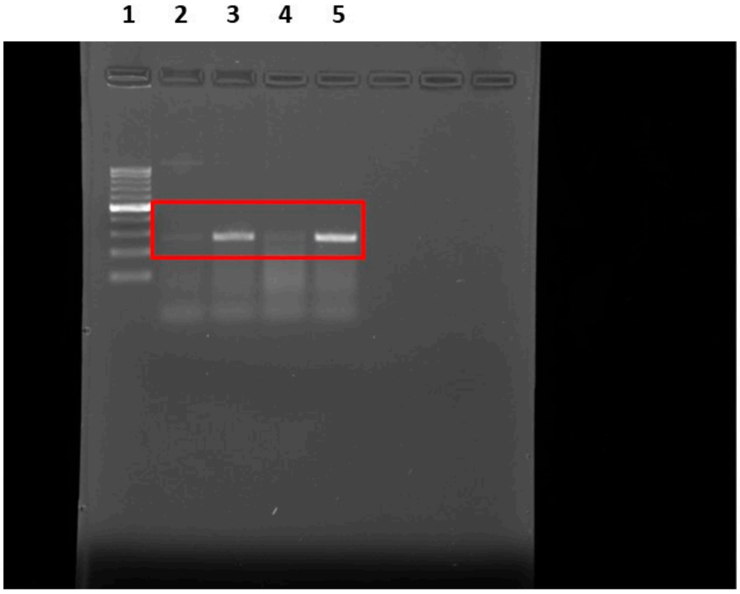

CAIX

1. Molecular weight DNA ladder
2. WM115, Normoxia, 48h
3. WM115, Hypoxia, 48h
4. WM266-4, Normoxia, 48h
5. WM266-4, Hypoxia, 48h

RT-PCR

Phosphofructo-2- kinase/fructose-2,6-biphosphatase 4

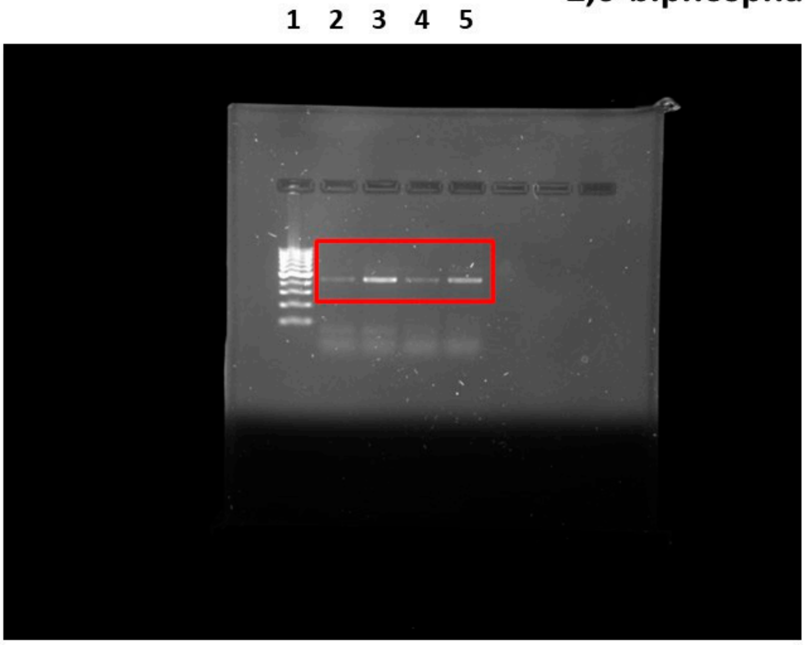

PFKFB4

1. Molecular weight DNA ladder
2. WM115, Normoxia, 16h
3. WM115, Hypoxia, 16h
4. WM266-4, Normoxia, 16h
5. WM266-4, Hypoxia, 16h

RT-PCR

Phosphofructo-2- kinase/fructose-2,6-biphosphatase 4

1 2 3 4 5

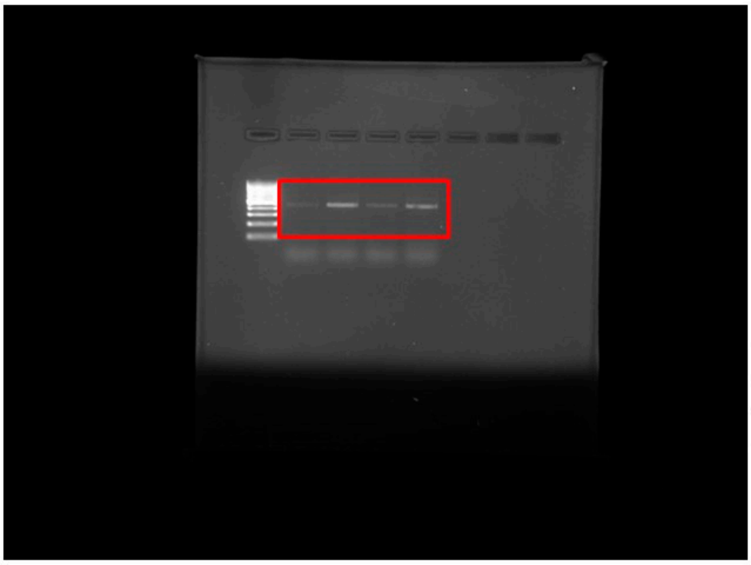

PFKFB4

1.Molecular weight DNA ladder
2. WM115, Normoxia, 24h
3. WM115, Hypoxia, 24h
4. WM266-4, Normoxia, 24h
5. WM266-4, Hypoxia, 24h

RT-PCR

Phosphofructo-2- kinase/fructose-2,6-biphosphatase 4

1 2 3 4 5

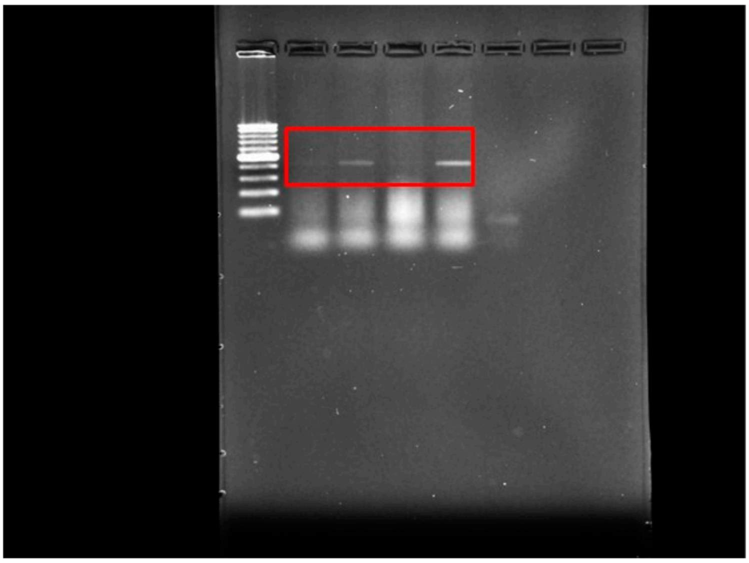

PFKFB4

1. Molecular weight DNA ladder
2. WM115, Normoxia, 48h
3. WM115, Hypoxia, 48h
4. WM266-4, Normoxia, 48h
5. WM266-4, Hypoxia, 48h

RT-PCR

3-Mercaptopyruvate sulfurtransferase

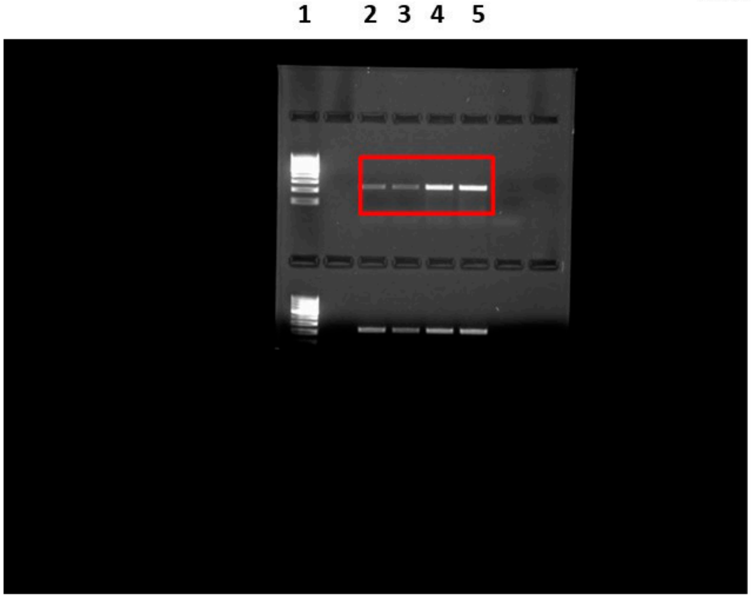

MPST

1. Molecular weight DNA ladder
2. WM115, Normoxia, 24h
3. WM115, Hypoxia, 24h
4. WM266-4, Normoxia, 24h
5. WM266-4, Hypoxia, 24h

RT-PCR

Cystathionine-β-synthase

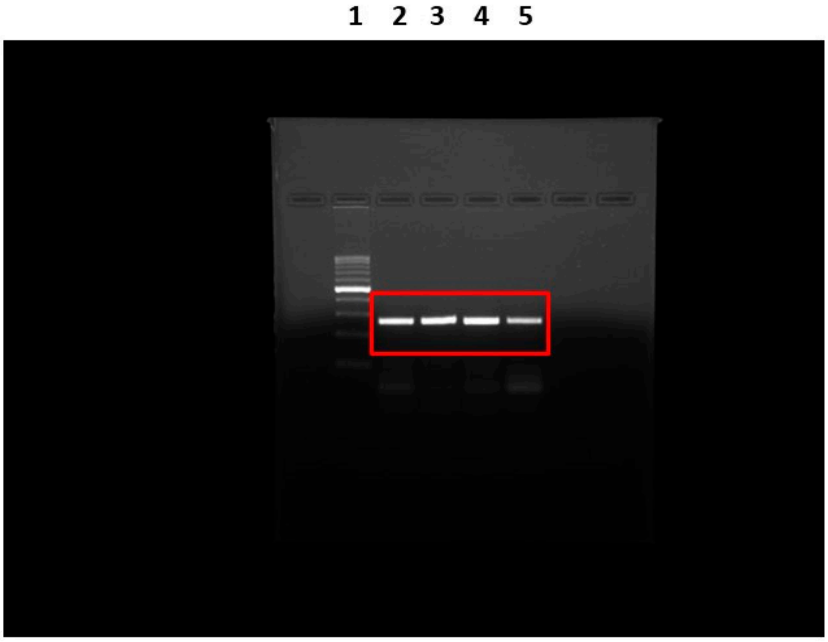

CBS

1. Molecular weight DNA ladder
2. WM115, Normoxia, 24h
3. WM115, Hypoxia, 24h
4. WM266-4, Normoxia, 24h
5. WM266-4, Hypoxia, 24h

RT-PCR

ɣ-Cystathionase

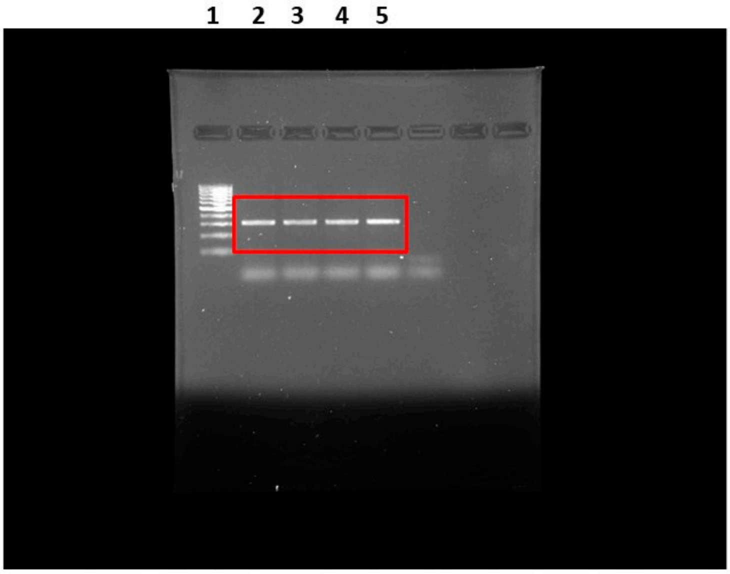

CTH

1. Molecular weight DNA ladder
2. WM115, Normoxia, 24h
3. WM115, Hypoxia, 24h
4. WM266-4, Normoxia, 24h
5. WM266-4, Hypoxia, 24h

RT-PCR

Thiosulfate sulfurtransferase

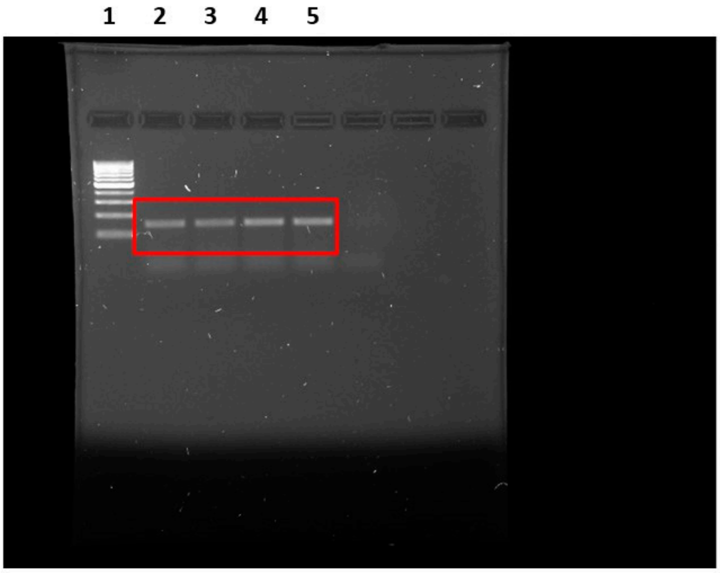

TST

1. Molecular weight DNA ladder
2. WM115, Normoxia, 24h
3. WM115, Hypoxia, 24h
4. WM266-4, Normoxia, 24h
5. WM266-4, Hypoxia, 24h

RT-PCR

Thioredoxin reductase 1

1 2 3 4 5

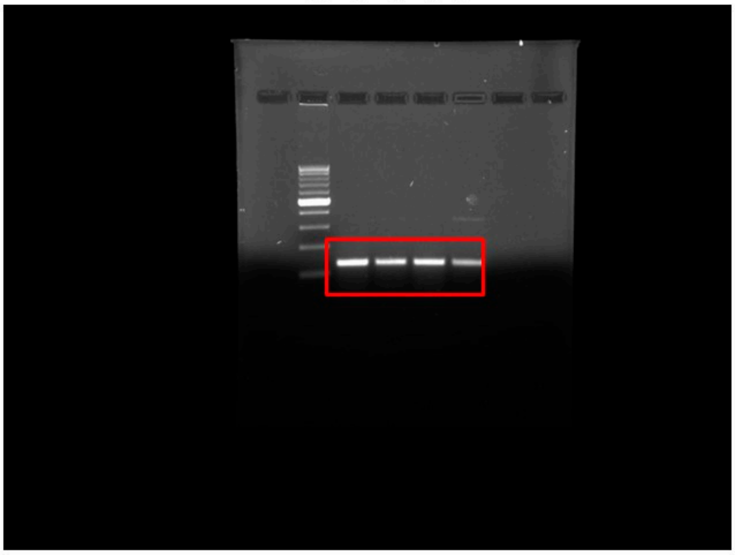

TXNRD1

1. Molecular weight DNA ladder
2. WM115, Normoxia, 24h
3. WM115, Hypoxia, 24h
4. WM266-4, Normoxia, 24h
5. WM266-4, Hypoxia, 24h

RT-PCR

Thioredoxin

1 2 3 4 5

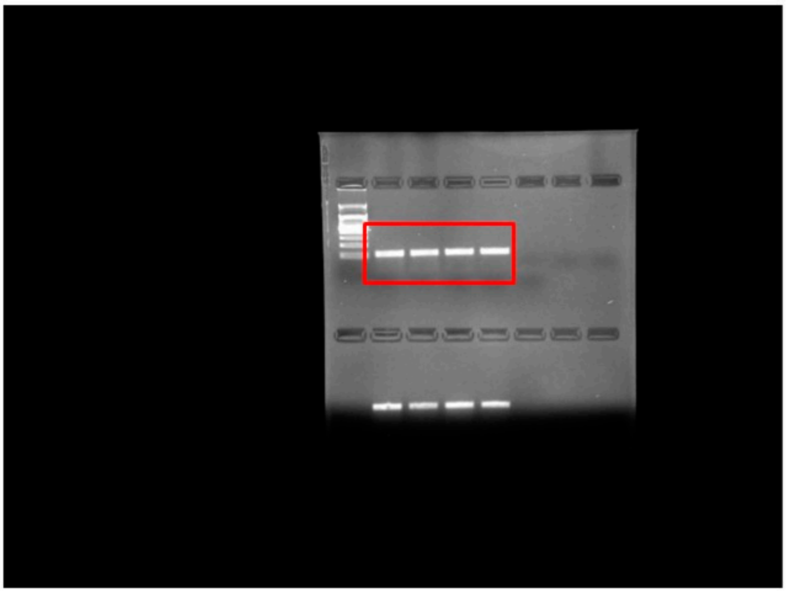

TRX

1. Molecular weight DNA ladder
2. WM115, Normoxia, 24h
3. WM115, Hypoxia, 24h
4. WM266-4, Normoxia, 24h
5. WM266-4, Hypoxia, 24h

RT-PCR

Glutathione peroxidase

1 2 3 4 5

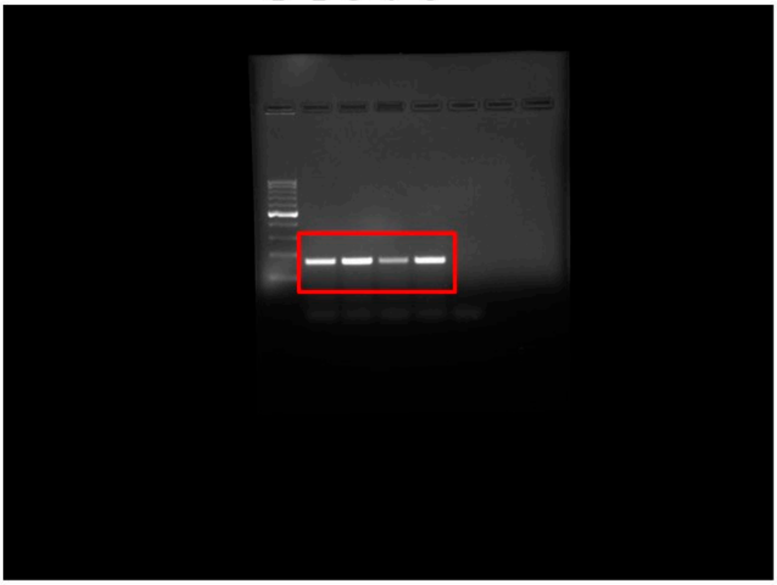

PGx

1. Molecular weight DNA ladder
2. WM115, Normoxia, 24h
3. WM115, Hypoxia, 24h
4. WM266-4, Normoxia, 24h
5. WM266-4, Hypoxia, 24h

WESTERN BLOT

Cystathionine-β-synthase

1 2 3 4 5 6 7 8 9 10

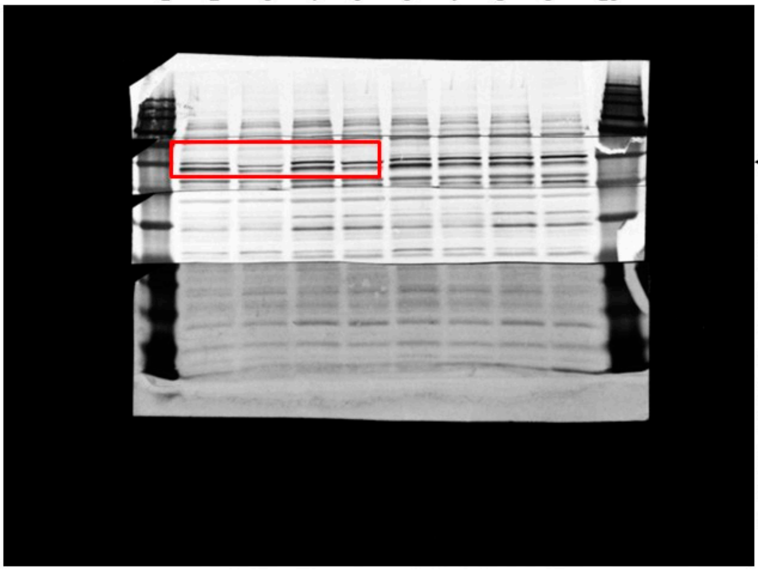

← CBS

1. Molecular weight standard
2. WM115, Normoxia, 24h
3. WM115, Hypoxia, 24h
4. WM266-4, Normoxia, 24h
5. WM266-4, Hypoxia, 24h
6. WM115, Normoxia, 48h
7. WM115, Hypoxia, 48h
8. WM266-4, Normoxia, 48h
9. WM266-4, Hypoxia, 48h
10. Molecular weight standard

WESTERN BLOT

3-Mercaptopyruvate sulfurtransferase

1 2 3 4 5 6 7 8 9 10

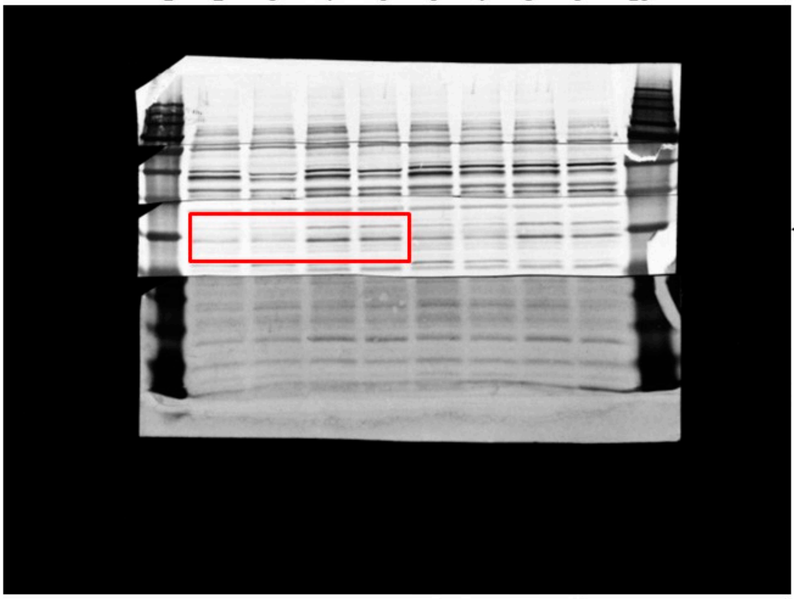

← MPST

1. Molecular weight standard
2. WM115, Normoxia, 24h
3. WM115, Hypoxia, 24h
4. WM266-4, Normoxia, 24h
5. WM266-4, Hypoxia, 24h
6. WM115, Normoxia, 48h
7. WM115, Hypoxia, 48h
8. WM266-4, Normoxia, 48h
9. WM266-4, Hypoxia, 48h
10. Molecular weight standar[illegible]

WESTERN BLOT

ɣ-Cystathionase

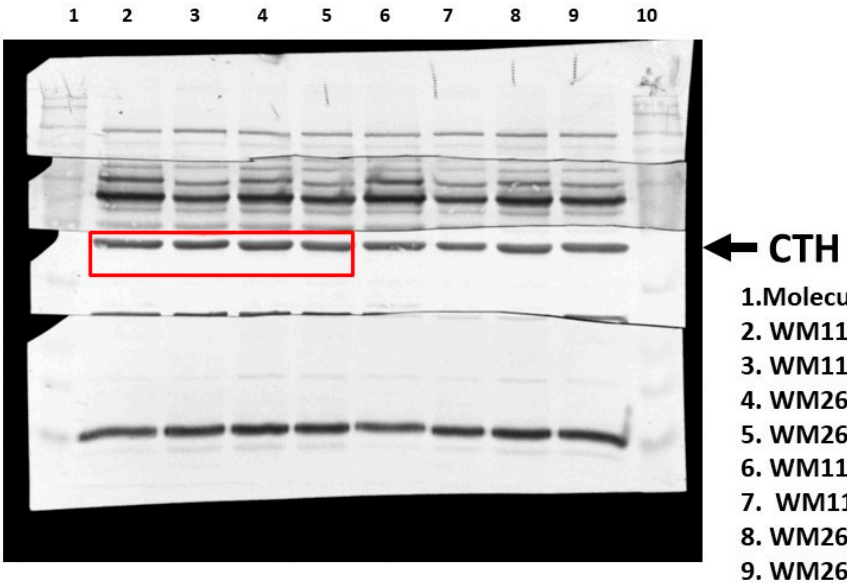

1.Molecular weight standard
2. WM115, Normoxia, 24h
3. WM115, Hypoxia, 24h
4. WM266-4, Normoxia, 24h
5. WM266-4, Hypoxia, 24h
6. WM115, Normoxia, 24h
7. WM115, Hypoxia, 24h
8. WM266-4, Normoxia, 24h
9. WM266-4, Hypoxia, 24h
10. Molecular weight standard

WESTERN BLOT

Thiosulfate sulfurtransferase

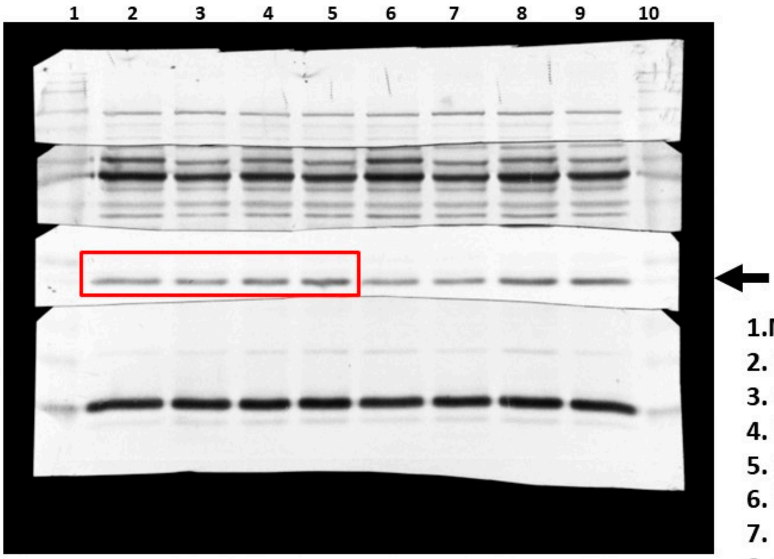

1.Molecular weight standard
2. WM115, Normoxia, 24h
3. WM115, Hypoxia, 24h
4. WM266-4, Normoxia, 24h
5. WM266-4, Hypoxia, 24h
6. WM115, Normoxia, 24h
7. WM115, Hypoxia, 24h
8. WM266-4, Normoxia, 24h
9. WM266-4, Hypoxia, 24h
10. Molecular weight standard

WESTERN BLOT

Thioredoxin reductase 1

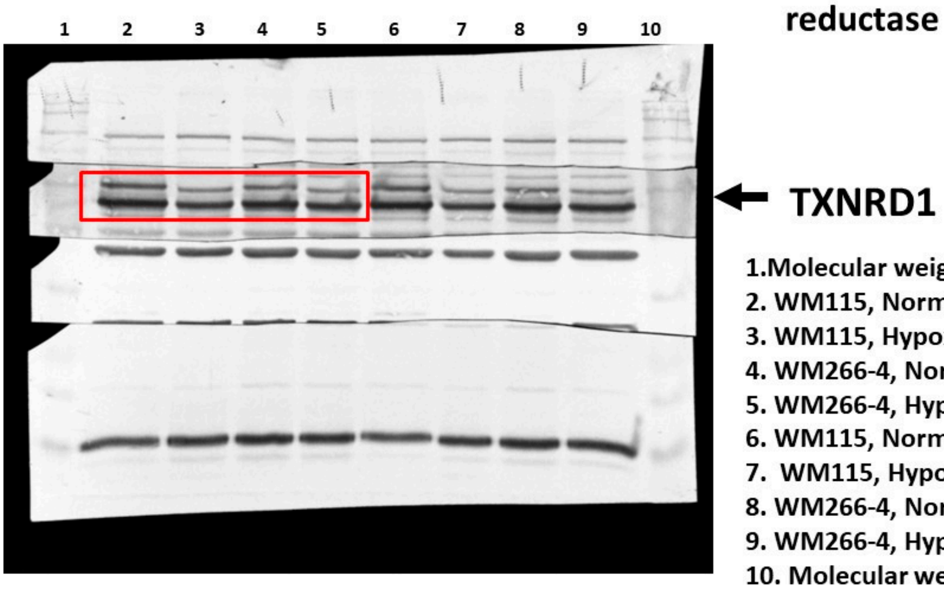

1.Molecular weight standard
2. WM115, Normoxia, 24h
3. WM115, Hypoxia, 24h
4. WM266-4, Normoxia, 24h
5. WM266-4, Hypoxia, 24h
6. WM115, Normoxia, 24h
7. WM115, Hypoxia, 24h
8. WM266-4, Normoxia, 24h
9. WM266-4, Hypoxia, 24h
10. Molecular weight standard

WESTERN BLOT

Superoxide dismutase 1

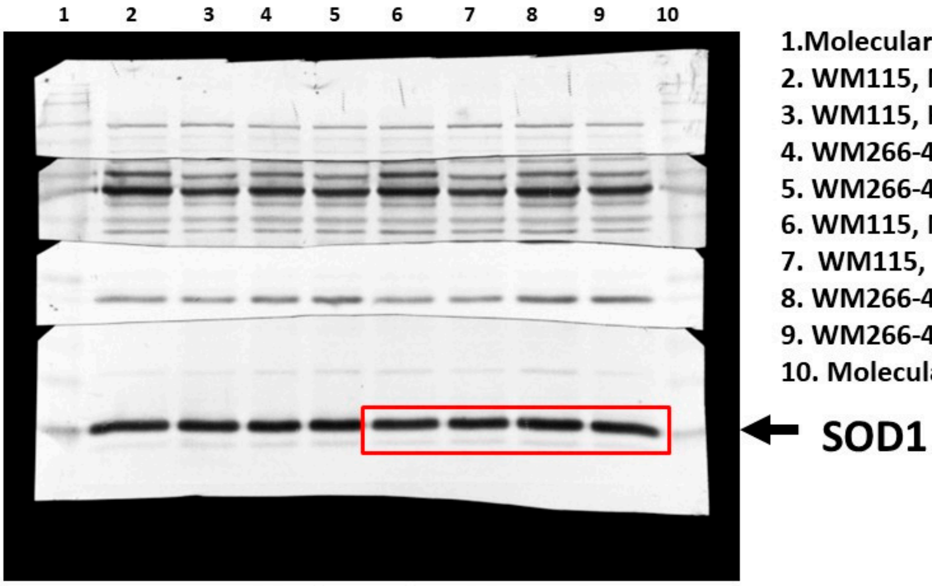

1.Molecular weight standard
2. WM115, Normoxia, 24h
3. WM115, Hypoxia, 24h
4. WM266-4, Normoxia, 24h
5. WM266-4, Hypoxia, 24h
6. WM115, Normoxia, 24h
7. WM115, Hypoxia, 24h
8. WM266-4, Normoxia, 24h
9. WM266-4, Hypoxia, 24h
10. Molecular weight standard

WESTERN BLOT

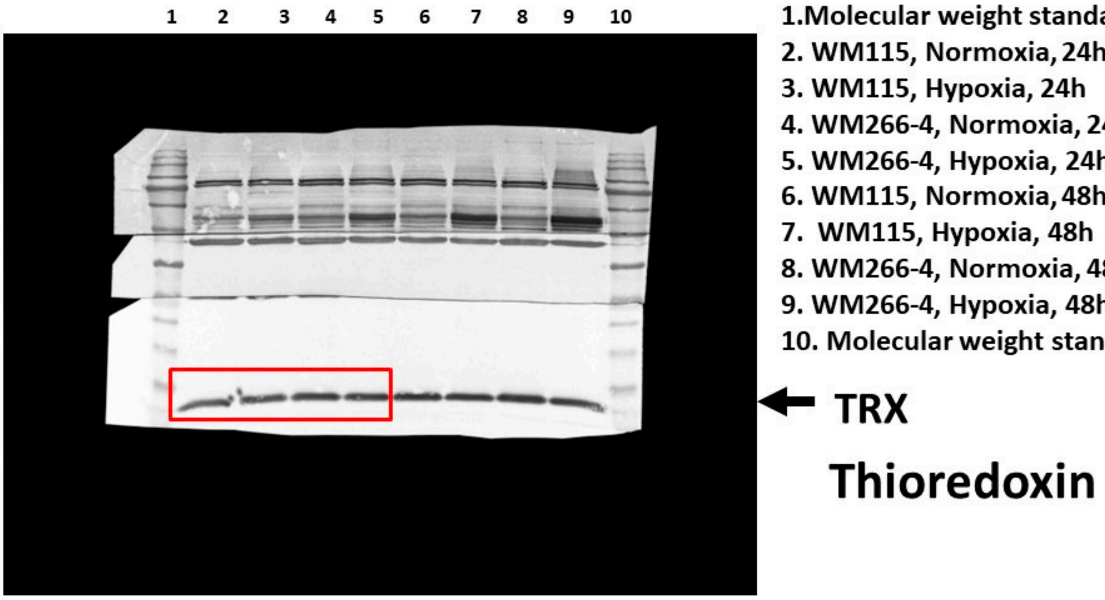

1.Molecular weight standard
2. WM115, Normoxia, 24h
3. WM115, Hypoxia, 24h
4. WM266-4, Normoxia, 24h
5. WM266-4, Hypoxia, 24h
6. WM115, Normoxia, 48h
7. WM115, Hypoxia, 48h
8. WM266-4, Normoxia, 48h
9. WM266-4, Hypoxia, 48h
10. Molecular weight standard

WESTERN BLOT

Carbonic anhydrase IX

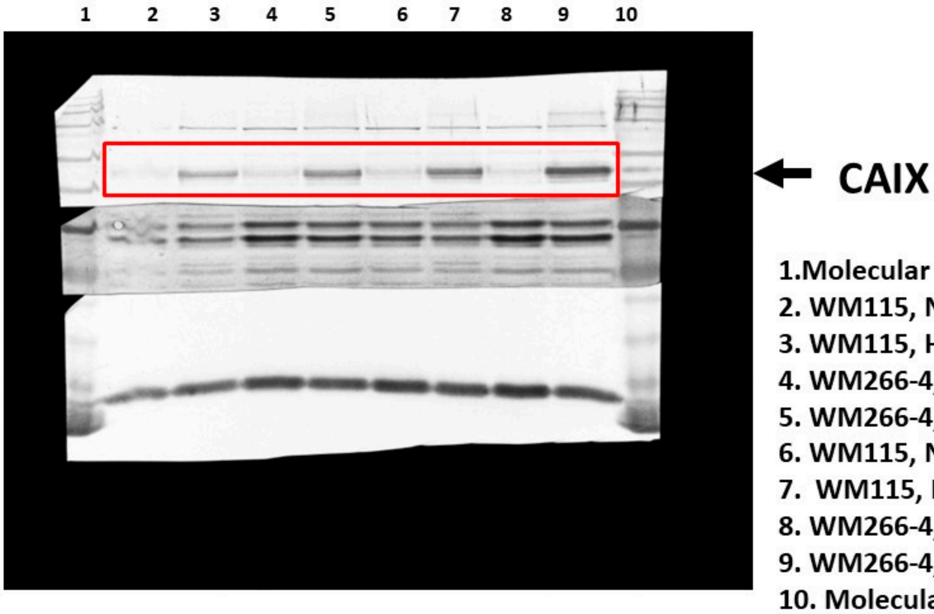

1.Molecular weight standard
2. WM115, Normoxia, 24h
3. WM115, Hypoxia, 24h
4. WM266-4, Normoxia, 24h
5. WM266-4, Hypoxia, 24h
6. WM115, Normoxia, 48h
7. WM115, Hypoxia, 48h
8. WM266-4, Normoxia, 48h
9. WM266-4, Hypoxia, 48h
10. Molecular weight standard

WESTERN BLOT

β-actin

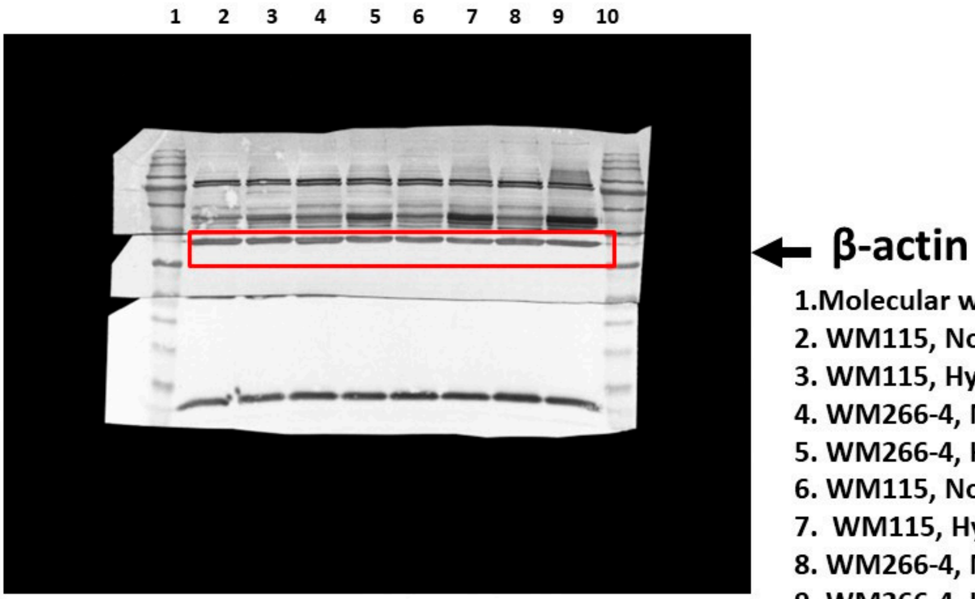

1.Molecular weight standard
2. WM115, Normoxia, 24h
3. WM115, Hypoxia, 24h
4. WM266-4, Normoxia, 24h
5. WM266-4, Hypoxia, 24h
6. WM115, Normoxia, 48h
7. WM115, Hypoxia, 48h
8. WM266-4, Normoxia, 48h
9. WM266-4, Hypoxia, 48h
10. Molecular weight standard

WESTERN BLOT

Carbonic anhydrase IX

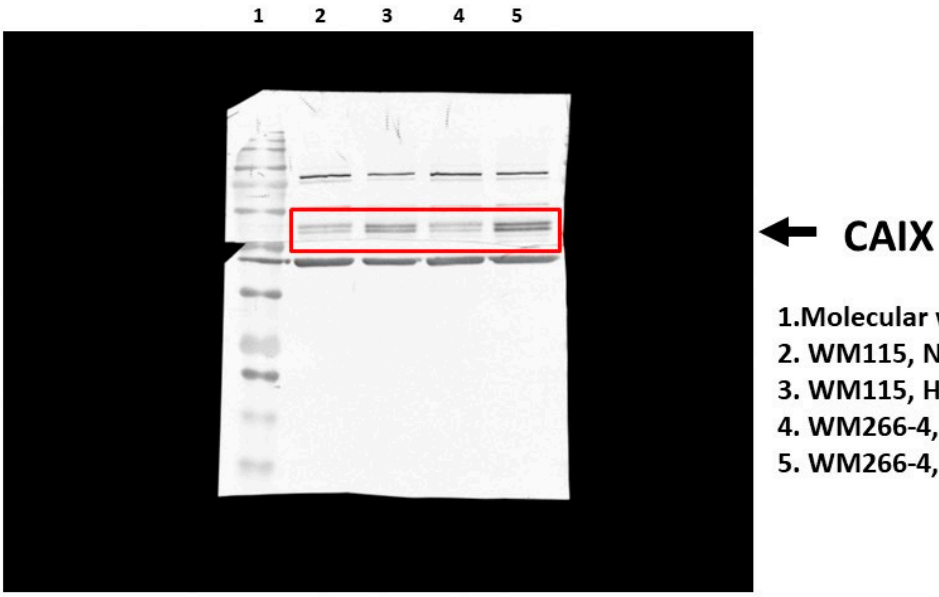

1.Molecular weight standard
2. WM115, Normoxia, 16h
3. WM115, Hypoxia, 16h
4. WM266-4, Normoxia, 16h
5. WM266-4, Hypoxia, 16h

WESTERN BLOT

β-actin

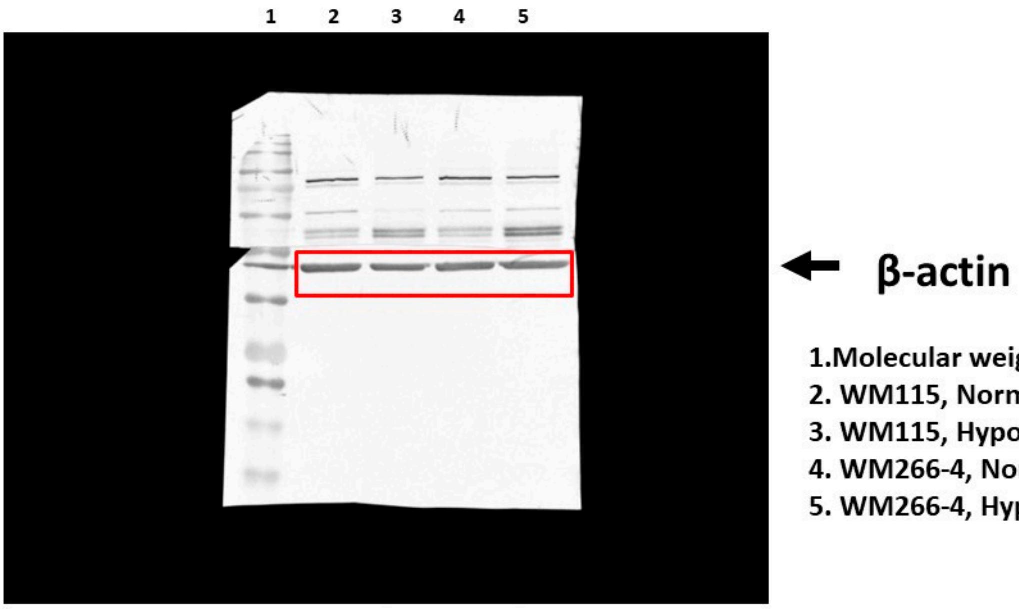

1.Molecular weight standard
2. WM115, Normoxia, 16h
3. WM115, Hypoxia, 16h
4. WM266-4, Normoxia, 16h
5. WM266-4, Hypoxia, 16h
